# Safe mobility, socioeconomic inequalities, and aging: A 12-year multilevel interrupted time-series analysis of road traffic death rates in a Latin American country

**DOI:** 10.1371/journal.pone.0224545

**Published:** 2020-01-07

**Authors:** Pablo Martínez, Daniela Contreras, Mónica Moreno

**Affiliations:** 1 CITIAPS, Universidad de Santiago de Chile, Santiago, Chile; 2 Escuela de Psicología, Facultad de Humanidades, Universidad de Santiago de Chile, Santiago, Chile; 3 Departamento de Psiquiatría y Salud Mental, Hospital Clínico Universidad de Chile, Santiago, Chile; 4 Instituto Milenio para la Investigación en Depresión y Personalidad (MIDAP), Santiago, Chile; 5 Núcleo Milenio para Mejorar la Salud Mental de Adolescentes y Jóvenes (Imhay), Santiago, Chile; University of Shanghai for Science and Technology, CHINA

## Abstract

As the resources for road safety in developing countries are scarce and unevenly distributed, vulnerable road users -such as the elderly- may be particularly at risk of road traffic deaths. To date, the impact of road safety measures over the rate of road traffic deaths in older adults (60 years or older), considering the within-country socioeconomic inequalities, has not been explored in developing nations. This study takes the Chilean case as an example -with its 2005 traffic law reform as one of the road safety measures investigated-, in which open data available from official national sources for all its 13 regions over the 2002–2013 period were used for a multilevel interrupted time-series analysis. A statistically significant secular reduction of the rates of road traffic deaths in the elderly population was found (incidence rate ratio [IRR] 0.95, 95% confidence interval [CI] 0.91 to 0.99), but no evidence for a significant intercept or slope change after the traffic law reform was observed. Regions with the highest number of traffic offenses prosecuted in local police courts had lower rates of road traffic deaths in older adults (IRR 0.95, 95% CI 0.90 to 1.00), and those regions in the third (IRR 1.61, 95% CI 1.16 to 2.25) and the fifth (IRR 1.66, 95% CI 1.08 to 2.54) quintiles of socioeconomic deprivation had higher rates of road traffic deaths in the elderly. Such findings strongly support the conceptualization of the road safety of seniors in developing countries as a social equity issue, with implications for the design of traffic regulations and road environments.

## 1 Introduction

City design at present emphasizes developing optimal and efficient mobility systems that have an impact on the wellbeing of people [1]. One of the negative consequences of land-use and transportation conditions in cities (i.e., heavily car-dominated infrastructure), is the increased risk of road traffic deaths [1]. Currently listed as the ninth cause of death, with a worldwide prevalence estimated at 2.5% [2], road traffic deaths are a major global public health problem.

In 2004, the World Health Organization and the World Bank issued a report that documented the need to address this serious global challenge, providing a synthesis of sound scientifically validated and successful road safety measures, and urging governments around the world to implement effective strategies to ensure road safety, to avoid driving under influence of alcohol, speeding, under-utilization of seat belts and child restraint system, poor road infrastructure, unsafe vehicle design, and under-implementation of road safety standards [3].

Influenced by these recommendations and after 12 years of public debate, in 2005, Chile adopted a comprehensive traffic law reform, expecting to reduce road traffic deaths [4]. The traffic law reform mandated the use of seat belts, and child restraint system in vehicles, and the use of helmets for motorcyclists and cyclists, forbade the use of cell phones by drivers, established higher fines for driving under the influence of alcohol, lowered the speed limit, delimited pedestrian traffic within cities, and introduced penalties for pedestrian offences, among others [4].

There is recent evidence suggesting that traffic fatality and injury rates among general and children populations in Chile may have been reduced by the introduction of the traffic law reform [5–6], and that large gains were observed when the enactment of the Chilean traffic law reform was followed by stronger police enforcement and supported by investments in road infrastructure, as it was demonstrated in the case of the general population [5]. However, research is lacking in such a vulnerable road user population as the older adults, which are differentially affected by chronic medical conditions and the use of medications [7–8], and age-related performance declines in driving or street crossing behaviors [9–10].

Chile’s rapidly aging population has meant that, during the first half of the 2010 decade, the proportion of elderly with valid driving license increased by 20 percent [11]. Although studies have shown that seniors are at increased risk of adverse outcomes after road injuries [12–13], no road safety plan specifically aimed at this population exist in Chile, and there is limited international evidence for a reduction of crash rates because of road safety awareness programs [14] or fitness to drive screening in the elderly [15].

Though aging and frailty partially explains the plight of this group of the population [8, 10], it also has been stressed that changes to road infrastructure and vehicle design are necessary to improve the traffic safety of the elderly [14], a challenge that need to be addressed by national authorities. Moreover, as older adults are particularly vulnerable to poverty [16], they may be economically forced to live in poor areas with inadequate roadway design, exposing them to excess risk of road traffic injuries [17].

As a large proportion of road traffic deaths affects poor and vulnerable road users, such as the elderly, road safety has been regarded as a social equity issue [3]. For instance, area-level socioeconomic disadvantage, whether measured through deprivation indicators or household (or neighborhood) income inequality, has been associated with a higher risk of death after a crash [18–19]; and significant between-country differences have been found, with developing nations having higher rates of road traffic deaths, affecting especially older pedestrians [20].

As aging population is growing globally, so does the older adult road users, and the road safety and mobility needs for the elderly [21], particularly those living in less privileged areas or less developed countries. However, few studies on the impact of area-level socioeconomic disadvantages on seniors’ risk of road traffic injury or death have been carried out, with a New Zealand study finding that younger age groups (vs. older adults) were more affected by this type of inequality [22].

Moreover, as the resources for road safety in developing countries are scarce [23] and unevenly distributed [24], the institutional capacity for the enforcement of traffic safety regulations in different geographical areas within these nations may be seriously affected, thus reducing the effectiveness of national road safety initiatives, rendering vulnerable road users defenseless. In this regard, the case of Chile, with an aging population, marked socioeconomic inequalities at the regional level, and important road safety measures implemented during the past decade, may be an interesting example for other developing nations.

This is the first study to examine the geographical and temporal associations of road safety measures, socioeconomic inequalities, and road traffic deaths in the elderly, as previous studies have performed pooled analysis over several years [22] or geographical areas [5–6], conflating between- and within-regional processes (or time-dependent and time-independent effects). This study aimed to evaluate the impact of a set of road safety measures (the Chilean traffic law reform, the number of traffic offenses prosecuted in local police courts, and investment in road infrastructure), over the rate of road traffic deaths in older adults (60 years or older), considering the socioeconomic inequalities in the 13 regions of Chile over a period of 12 years (2002–2013).

## 2 Materials and methods

### 2.1 Data sources

Open data available from official national sources were obtained. These sources were:

- The National Institute of Statistics (INE), used to gather the historical series of the consumer price index, population and labor statistics, number of vehicles, and the number of traffic offenses prosecuted in local police courts.
- The Ministry of Social Development (MIDESO), responsible for the design and implementation of social policies, consulted for estimates of population’s situation of poverty, education, and income.
- The Secretariat for the Prevention of Crime (SPD), of the Ministry of the Interior and Public Security, consulted for police statistics on violent and property crimes.
- The Ministry of Public Works (MOP), to incorporate the historical series of investment in road infrastructure.
- The National Road Safety Commission (CONASET, for its name in Spanish), the national agency responsible for the reduction of road traffic crashes and their consequences, was consulted for statistics of road traffic deaths.

These data were consolidated in a time-series from year 2002 to 2013 for each of the 13 administrative divisions (regions) of Chile. Due to biennial and/or triennial recurrence of data from the MIDESO, interpolation methods with cubic splines were used to estimate missing values of the period studied. The database had 156 observations each per year and region of the study.

### 2.2 Study population

Older adults–aged 60 or older–without distinction by sex or type of road traffic user, who died or not during the 24 hours following a road traffic crash, according to the road traffic deaths registered by CONASET.

### 2.3 Study variables

All the study variables were repeated measures observed at time *i* within region *j*.

#### 2.3.1 Dependent variable

Counts of road traffic deaths in older adults (*y*_*ij*_).

#### 2.3.2 Time-Varying Covariates (TVCs)

f Time (*x*_1*ij*_): As the Chilean traffic law reform was introduced in late 2005, the year 2006 was considered as the implementation year. Thus, time was centered on the year 2006.
g Traffic law reform (*x*_2*ij*_): dummy covariate for observations recorded the years after the main road safety measure–i.e., the Chilean traffic law reform- was homogeneously introduced in all regions of Chile, versus those observations recorded the years before the intervention. In this regard, year 2006 was censored, as it was assumed that the traffic law reform was implemented over this year.
h Traffic offenses (*x*_3*ij*_): number of traffic offenses prosecuted in local police courts for every 100,000 inhabitants; TVC used to assess the institutional capacity to ensure law enforcement as a road safety measure.
i Investment in road infrastructure (*x*_4*ij*_): investments in road infrastructure, in hundreds of dollars per capita adjusted by the consumer price index; TVC used to evaluate road network improvement as a road safety measure. Table 1 provides a summary of the major investments in road infrastructure during the study period.
j Quintiles of deprivation (*x*_5*ij*_,*x*_6*ij*_,…,*x*_9*ij*_,): dummy covariates for a synthetic measure of socioeconomic inequalities–i.e., deprivation index (*w*_1*ij*_)–in the regions of Chile, with higher quintiles representing more deprivation; an explanation is provided in the following subsection.
k Vehicles per capita (*x*_10*ij*_): number of vehicles per 10,000 inhabitants.
l Ageing index (*x*_11*ij*_): number of people aged 60 years and over per hundred persons under age 15.

**Table 1:** Major investments in road infrastructure in Chile, 2002–2013.

Major Public Road Works
1. Installation of physical elements such as traffic lights, roundabouts, pedestrian crossings, overpasses and public lighting on busy roads. 2. Segregation of the different types of transportation and road users. 3. Extensions of existing roads. 4. New route layouts to support private and cargo transport. 5. New solutions of public transport (exclusive corridors, differentiated roads, new services). 6. Conservation work on road networks. 7. Activation, improvement, and expansion of routes.

#### 2.3.3 On the construction of the deprivation index

Four socioeconomic variables were grouped in a deprivation index (*w*_1*ij*_) to obtain a simple measure of socioeconomic inequalities in the regions of Chile. This was based on reputable variables for measuring socioeconomic inequalities in health [25–26], considering the socioeconomic predictors of risk of road traffic injury [18], together with the data available in national sources of information. This index is composed of the following variables:

- Individual poverty (*w*_2*ij*_): percentage of people with an autonomous per capita family income below the cost of two basic food baskets.
- Insufficient schooling (*w*_3*ij*_): percentage of people 18 years and over who have less than 12 years of schooling.
- Unemployment (*w*_4*ij*_): percentage of the working age people who are jobless, were looking for jobs within the last 4 weeks, and are available for work.
- Crimes (*w*_5*ij*_): number of violent and property crimes per 100,000 inhabitants.

These variables were standardized by transformation to Z-scores, and then the deprivation index (*w*_1*ij*_) was obtained by simple sum, which was subsequently divided into quintiles (*x*_5*ij*_,*x*_6*ij*_,…,*x*_9*ij*_,). Table 2 reports descriptive statistics for the deprivation index (*w*_1*ij*_) and the variables that compose it.

**Table 2:** Description of the deprivation index and related variables.

Variables	Notation	Mean	SD	Min	Max
Deprivation index	(*w*_1*ij*_)	0.00	2.0	-5.65	5.42
Individual poverty	(*w*_2*ij*_)	22.30	6.79	9.43	39.57
Insufficient schooling	(*w*_3*ij*_)	63.61	6.14	50.74	75.32
Unemployment	(*w*_4*ij*_)	4.32	1.39	1.25	8.17
Crimes	(*w*_5*ij*_)	2428.20	509.65	1114.01	3766.12

SD, standard deviation; Min, minimum; Max, maximum.

### 2.4 Statistical analysis

#### 2.4.1 General analytical framework

This study worked with annual time-series (2002–2013) for each region of Chile, in which annual measurement occasions *i* –at level 1- were nested within regions *j* –level 2 units–prompting a multilevel modeling strategy for the analysis of longitudinal data, an analytical approach which provides the unique opportunity to examine between- and within-regional processes [27].

#### 2.4.2 Exploratory analyses

As a first step, the overall descriptive statistics and trends in rates of road traffic deaths in older adults (per 10,000 vehicles), at both the national and regional levels, were calculated with due emphasis on the pre-post comparison of the traffic law reform. Prior to the multilevel modeling of the data, the variance inflation factor (VIF) was verified in candidate covariates to study multicollinearity [28], as collinearity (VIF>10) was detected between time (*x*_1*ij*_) and trends in ageing index (*x*_11*ij*_), this latter covariate was dropped from the model-building process, resulting in a VIF average of 2.07, and no covariate with a VIF greater than 10.

#### 2.4.3 Disaggregation of between–and within–regional effects

The use of composite or conflated effects may hinder the exploration of between- and within-regional effects. Therefore, before model construction, the disaggregation of effects was warranted and done in accordance with Wang, & Maxwell [29]. To this end, the region-specific mean of a given TVC was subtracted from the raw TVC score obtained at each occasion, this approach is known as person-mean centering (PMC). Consider as an example the case of traffic offenses *x*_3*ij*_, after applying the PMC strategy, two types of covariates were produced: 1) ${\bar{x}}_{3.j}$, the region-specific mean for region *j*, a level 2 covariate with no variation within regions; and, 2) ${\dot{x}}_{3ij}$, a level 1 covariate with no variation between regions, centered around the region-specific mean ${\bar{x}}_{3.j}$. Furthermore, the region-specific mean ${\bar{x}}_{3.j}$ was grand-mean centered (GMC) by subtracting the grand mean ${\bar{x}}_{3..}$ from it, obtaining ${\ddot{x}}_{3.j}$.

Time *x*_1*ij*_, and the Chilean traffic law reform *x*_2*ij*_, were not subjected to PMC, as these covariates represented exclusively within-region variation. PMC was also not applied to vehicles per capita *x*_10*ij*_, since this was used as an offset covariate.

#### 2.4.4 Generalized linear mixed model

Regarding the model-building process, the Poisson distribution for rare events has been regarded as the standard for modeling crash-related data [30]. However, the data was over-dispersed–i.e., the variance exceeds the mean of the road traffic death counts-, such a common characteristic of crash-related data has been usually handled with the Negative Binomial distribution [30]. In this study, and given the analytical framework, exponentiated regression coefficients (i.e., incidence rate ratios, IRR) with their 95% confidence intervals (95% CI) were estimated using generalized linear mixed models with a log-link function and a Negative Binomial distribution, through the meglm procedure in Stata 14.0 [31]. To fit this model, the covariate vehicles per capita *x*_10*ij*_ was considered as a varying exposure rate, introduced through log transformation *ln* (*x*_10*ij*_)–an offset covariate- to model rates of road traffic deaths in older adults per 10,000 vehicles. As a proxy for regional level variation explained, the proportional reductions in the variance component relative to the basic model (see section 2.4.5) were computed.

#### 2.4.5 Interrupted time-series analysis

As the introduction of the Chilean traffic law reform may be considered a natural experiment, interrupted time-series analysis was considered to adequately assess the impact of this intervention [32]. For this reason, an interaction term between time and the Chilean traffic law reform *x*_1*ij*_*x*_2*ij*_ was added to the model, allowing for the direct estimation of both the immediate and the gradual change in the outcome as a product of the intervention, accounting for pre-intervention trends [32]. This was the basic model, which was sequentially adjusted for level 1 and level 2 covariates, respectively.

#### 2.4.6 Analytical multilevel model

According to the previously stated specifications, the analytical multilevel model is displayed in the following equation.

$$ln\;\left(\frac{\mu_{ij}}{x_{10ij}}\right)={(\beta }_{0}+\zeta_{0j})+\beta_{1}x_{1ij}+\beta_{2}x_{2ij}+\beta_{3}x_{1ij}x_{2ij}+\beta_{4}{\dot{x}}_{3ij}+\beta_{5}{\dot{x}}_{4ij}+\beta_{6}{\dot{x}}_{6ij}+\beta_{7}{\dot{x}}_{7ij}+\beta_{8}{\dot{x}}_{8ij}+\beta_{9}{\dot{x}}_{9ij}+\beta_{10}{\ddot{x}}_{3.j}+\beta_{11}{\ddot{x}}_{4.j}+\beta_{12}{\ddot{x}}_{6.j}+\beta_{13}{\ddot{x}}_{7.j}+\beta_{14}{\ddot{x}}_{8.j}+\beta_{15}{\ddot{x}}_{9.j}+\varepsilon_{ij}$$

In Eq 1, the expected number of road traffic deaths *μ*_*ij*_ at occasion *i* within region *j*, was divided by the exposure *x*_10*ij*_, in a log-linear model with intercept *β*_0_, region-specific random intercept *ζ*_0*j*_ to account for within-region dependence, and explicit error term *ε*_*ij*_ to model over-dispersion. Following the conventions described in the previous sections, the first three coefficients after the random-effects term were the basic components of the interrupted time-series analysis, coefficients for PMC ${\dot{x}}_{ij}$ covariates represented within-region effects (level 1), and coefficients for GMC ${\ddot{x}}_{.j}$ covariates were between-region effects (level 2).

## 3 Results

### 3.1 Exploratory analyses

During the study period, the population of older adults (aged 60 or older) in Chile grew by 30.94%, representing 2,482,768 people and 115,252 road traffic crashes were recorded, which caused 3,822 road traffic deaths in elderly road users. The descriptive statistics for the variables included in the study are presented in Table 3.

**Table 3:** Overall summary statistics.

Variables	Mean	SD	Min	Max
***Dependent variable***				
Counts of road traffic deaths in older adults	24.50	23.71	0.00	110.00
***Pure within-region covariates***				
Time	1.50	3.46	-4.00	7.00
Chilean traffic law reform	0.58	0.49	0.00	1.00
***PMC ${\dot{x}}_{ij}$ covariates***				
Traffic offenses	7.53	1.49	2.01	12.32
Investment in road infrastructure	1.12	0.47	-0.12	4.06
Deprivation index	2.99	1.42	1.00	5.00
***GMC ${\ddot{x}}_{.j}$ covariates***				
Traffic offenses	-0.00	2.54	-2.88	4.99
Investment in road infrastructure	-0.00	0.87	-0.96	2.51
Deprivation index	2.85	1.41	1.00	5.00
***Offset covariate***				
Log of vehicles per capita	2.60	1.01	0.20	5.15

SD, standard deviation; Min, minimum; Max, maximum; PMC, person-mean centered; GMC, grand-mean centered.

**Fig 1:**
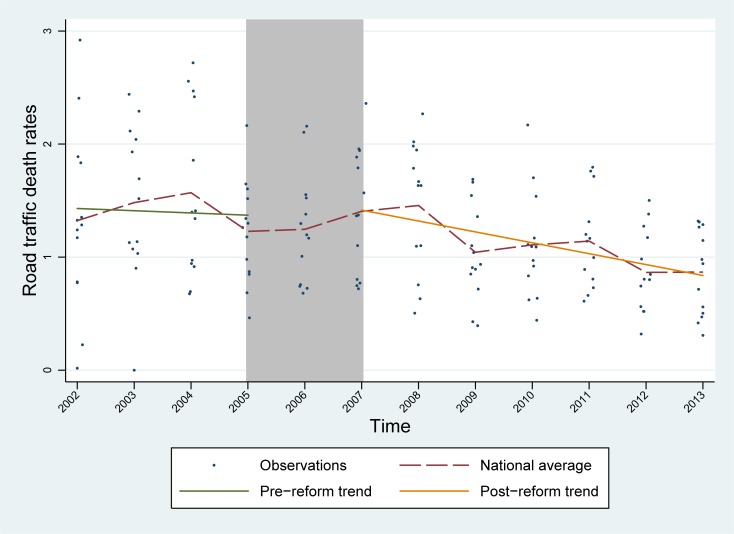
Average national rates of road traffic deaths in elderly over study period (2002–2013), with pre- and post-traffic law reform trends.

Fig 1 shows the average national rates of road traffic deaths in elderly over time, the crude pre- and post-intervention trends, with the shaded area representing the implementation period. An overall decreasing trend in the rates of road traffic deaths in elderly road users was observed, from 1.32 deaths per 10,000 vehicles in 2002 to 0.87 deaths per 10,000 vehicles by 2013. Furthermore, comparisons of the pre- and post-traffic law reform periods (i.e., 2002–2005, and 2007–2013, respectively), revealed a reduction in the outcome of approximately 20% in the aged population ($=\frac{\left(1.13-1.40\right)}{1.40}\times 100$) at the national level. Although the rates of road traffic deaths for the study population increased after the intervention in the regions of Tarapacá (20.86% $=\frac{\left(1.09-0.86\right)}{0.86}\times 100$), Atacama (2.79% $=\frac{\left(1.22-1.19\right)}{1.19}\times 100$), and Aysén ($105.78\%=\frac{\left(1.05-0.51\right)}{0.51}\times 100$), a basic pre-post analysis suggested that these differences were not significant (p-values>0.2).

### 3.2 Generalized linear mixed model

The generalized linear mixed models for rates of road traffic deaths in older adults per 10,000 vehicles are presented in Table 4. In the basic model (model 1), the fixed effects coefficients indicate that a statistically significant secular reduction in the rates of road traffic deaths in the elderly population has occurred over the entire study period (‘time’ covariate, IRR 0.94, 95% CI 0.91 to 0.98, p-value = .007), while changes in the intercept or slopes were minimal and did not reach statistical significance.

**Table 4:** Generalized linear mixed models for rates of road traffic deaths in older adults per 10,000 vehicles.

Covariates	Model 1^a^	Model 2^b^	Model 3^c^
	IRR (95% CI)	IRR (95% CI)	IRR (95% CI)
**Fixed part**			
Constant	1.24* (1.01, 1.52)	1.36 (0.94, 1.95)	1.01 (0.67, 1.53)
***Within-region effects***			
Time	0.94** (0.91, 0.98)	0.95* (0.91, 0.99)	0.95* (0.91, 0.99)
Traffic law reform	1.09 (0.94, 1.27)	1.11 (0.95, 1.29)	1.11 (0.95, 1.29)
Time × Traffic law reform	0.99 (0.94, 1.04)	0.98 (0.93, 1.03)	0.98 (0.93, 1.03)
Traffic offenses		0.99 (0.95, 1.03)	0.99 (0.95, 1.03)
Investment in road infrastructure		1.01 (0.86, 1.19)	1.01 (0.86, 1.19)
2^nd^ deprivation quintile		1.02 (0.89, 1.16)	1.02 (0.90, 1.17)
3^rd^ deprivation quintile		0.93 (0.82, 1.07)	0.94 (0.82, 1.08)
4^th^ deprivation quintile		0.95 (0.82, 1.10)	0.96 (0.83, 1.10)
5^th^ deprivation quintile		0.97 (0.83, 1.13)	0.97 (0.83, 1.13)
***Between-region effects***			
Traffic offenses			0.95* (0.90, 1.00)
Investment in road infrastructure			0.94 (0.81, 1.10)
2^nd^ deprivation quintile			1.29 (0.90, 1.85)
3^rd^ deprivation quintile			1.61** (1.16, 2.25)
4^th^ deprivation quintile			1.39 (0.98, 1.97)
5^th^ deprivation quintile			1.66* (1.08, 2.54)
**Random part**			
Constant	0.10 (0.04)	0.10 (0.04)	0.02 (0.01)
Proportional reduction in variance	-	-0.10%	76.46%

IRR, incidence rate ratio (fixed part of the models); 95% CI, 95% confidence interval.

Data for the random part of the models are variance (standard error).

^a^Model 1, basic model (time + traffic law reform + time × traffic law reform).

^b^Model 2, model 1 + person-mean centered ${\dot{x}}_{ij}$ covariates.

^c^Model 3, model 2 + grand-mean centered ${\ddot{x}}_{.j}$ covariates.

*p-value > .05.

**p-value > .01.

For the construction of model 2, the PMC ${\dot{x}}_{ij}$ covariates were added to the basic model, producing a negligible proportional increase in the variance component at the regional level (0.10%). Except for the secular trend described earlier for the model 1, there were no statistically significant within-region effects. In addition, the step-by-step introduction of the PMC ${\dot{x}}_{ij}$ covariates did not generate changes in the coefficients of the fixed or random part of the model.

For the full model (model 3), the addition step-by-step of the GMC ${\ddot{x}}_{.j}$ covariates, caused in a substantial reduction in the variance component at the regional level (76.46%). As a result, the sequential inclusion of the between-region effects of road safety measures and socioeconomic inequalities (measured through deprivation quintiles), decreased the variance component at the higher level by 54.22% ($=\frac{\left(0.10-0.05\right)}{0.10}\times 100$) and 48.59% ($=\frac{\left(0.05-0.02\right)}{0.05}\times 100$), respectively.

As for the fixed effects part of the model 3, no major changes in the within-region effects in comparison to model 2 were found. Also, the addition of GMC ${\ddot{x}}_{.j}$ covariates produced significant IRRs for traffic offenses, and socioeconomic inequalities. In this regard, the regions with a greater number of traffic offenses had lower rates of road traffic deaths in the study population (IRR 0.95, 95% CI 0.90 to 1.00, p-value = .047). Complementarily, the existence of a social gradient in the rates of road traffic deaths was confirmed: the higher the socioeconomic deprivation at the regional level, the higher the rates of road traffic deaths in seniors; specifically, regions belonging to the third and fifth quintile of deprivation had 1.61 (95% CI 1.16 to 2.25, p-value = .005) and 1.66 (95% CI 1.08 to 2.54, p-value = .019) times higher rates of road traffic deaths in comparison to the least socioeconomically deprived regions (first quintile), respectively.

## 4 Discussion

### 4.1 Findings and explanations

Road traffic death rates in the Chilean older adult population are decreasing over time without statistically significant changes attributable to a traffic law reform aimed at the general population. In addition, differences between regions in the institutional capacity to ensure law enforcement (i.e., number of traffic offenses) and in the levels of socioeconomic deprivation importantly affected the rates of road traffic deaths in the elderly. Such findings strongly support the conceptualization of the road safety of seniors in developing countries as a social equity issue.

The secular reduction of road traffic death rates reported in the Chilean aged population has also been observed at the international level [13]. Among other factors, these downward trends have been attributed to healthier and more active older adults who perform better in motorized transport systems of the developed world [13]. The Chilean Survey on Quality of Life in Old Age seems to support this explanation, reporting an increase in the rates of older adults with active lifestyle [33], suggesting that risk factors for road traffic deaths in this population (e.g., cognitive decline and frailty) may have been reduced over the study period [34].

The failure of the traffic law reform to decrease the rates of road traffic deaths in the study population may reflect a road safety measure designed to address the needs of the average road user, neglecting the requirements for the safe mobility of older adults [34]. As seniors have different capabilities [14], traffic regulations must be comprehensive enough to include or reinforce provisions related to pedestrians’ right-of-way and crossing times, or to the lowering of speed limits [35–36], areas not covered by the Chilean traffic regulation of 2005. In general, legal frameworks to ensure safe mobility for the elderly must be aimed at making the roads more amenable to the aging population [35].

In a very similar way, the lack of a significant association between investments in road infrastructure and the study outcome may be a symptom of the roadway environment being changed throughout the country over the past decades without considering the physical and cognitive abilities of older adults [9, 14]. Although an earlier study suggested that these investments indirectly reduced serious injuries in the Chilean general population [4], national and regional policies that contribute to sustainable and safe environments for all road users are required. In the case of the elderly, some of the roadway design enhancement that are recommended in the literature include the use of offset turn lanes, and the appropriate size and placement of street signs [14].

The analyses confirmed the uneven distribution of the resources to ensure law enforcement between the regions of Chile, as regional differences in the number of traffic offenses processed in local police courts accounted for rates of road traffic deaths in the elderly. As implied by a previous study in the country’s general population, the allocation of these resources was important in the reduction of road traffic deaths and injuries [37], constituting itself in a fundamental mean to promote behavioral change. In line with international evidence, these results show that legislative measures alone may not be enough to achieve gains in road user safety, requiring its adequate enforcement to ensure compliance [3, 38].

One of the main findings of this study was the existence of a social gradient in the rates of road traffic deaths in older adults at a regional-level. As Chile is faced with an aging population and marked within-country socioeconomic inequalities, the social stratification in road traffic deaths seems not to be decreasing with the passing of age, contradicting the results of studies carried out in developed countries [39–41]. Thus, like what has been described for the social determinants of road traffic injuries and deaths in the pediatric population [18], socioeconomically deprived environments may expose seniors to an increased risk to their safety at roads.

These vulnerable areas could offer few alternatives to secure mobility and visibility of vulnerable road users (including older adults) [42], exposing both pedestrians and drivers of this age group to roads with a significant volume of traffic, areas with little traffic speed regulation or poorly designed intersections [18, 42], hindering the reaction and/or decision-making capacity in these subjects [9–10]. This is complemented by the propensity to exhibit unsafe behaviors among elderly road users living in socially deprived areas [43], and the reduced availability of quality health services for emergency attention after a road traffic crash in these areas [44].

### 4.2 Strengths and limitations

This study merged official datasets from multiple government agencies to obtain regional-level data for road safety measures and socioeconomic covariates for the build-up of a time series that spanned over a decade. The sound socioeconomic covariates included were grouped in a deprivation index, which was created in accordance with previously described methods in the literature [25]. Moreover, the disaggregation of effects (i.e., the differentiation of PMC and GMC covariates) and the use of a multilevel modelling approach allowed to explicitly examine between- and within-regional (i.e., time-dependent) processes [27, 29]. In addition, the interrupted time-series framework permitted the estimation of immediate and delayed changes in the outcome as a result of the analyzed intervention (the Chilean traffic law reform), after adjusting for secular trends [32]. The scant literature on time series analysis to evaluate the impact of road safety measures on the rates of road traffic injuries or deaths in the elderly have not considered all these details and have been carried out in developed countries [45–47].

This study had several shortcomings. The spatial unit of analysis used was the highest administrative division of the country (i.e. regions), representing a considerable volume of population and an important level of heterogeneity in comparison to smaller administrative divisions (e.g., provinces, or municipalities). Thus, differences between provinces or municipalities may be of greater interest to the development of local interventions, as these units of analysis would consider proximal area-level conditions.

Complementarily, the CONASET database had some limitations: (a) road traffic deaths were recorded within the first 24 hours of the crash, which may have led to an underestimation of the actual death counts, although a previous study found no differences after comparing the hospital discharge registry with the police database [45]; (b) individual anonymized records lacked information on socioeconomic status, thus adjustment for individual-level socioeconomic disadvantage was not possible. Furthermore, though data for road traffic deaths were recorded daily, making possible the analysis of monthly time series for example, the INE, SPD, and MOP datasets mostly contained annual variations. In addition, the biennial or triennial character of the MIDESO database required the use of interpolation methods to estimate missing data points, which may have been a major source of bias.

### 4.3 Unanswered questions and future research

More research is needed on the role of road safety measures and socioeconomic inequalities in rates of road traffic deaths in the elderly, especially from developing countries. Some of the possible explanations offered for the study results require further confirmation (e.g., do reductions in cognitive decline and frailty over the 2002–2013 period effectively accounted for the downward trends in the outcome measure?), must consider analyses by important covariates (e.g., sex, type of road user, rural/urban areas, among others), and use different measures of risk-exposure (e.g., mileage, travel time, traffic density). Also, it remains to be explored whether there are differential impacts of road safety measures across levels of regional socioeconomic deprivation, such an approach would be of great interest for the implementation of equity-based policies in road safety, and to reduce the gap between socially deprived areas and the more affluent ones.

Complementarily, in view of the study limitations, future research should focus on: (a) smaller units of spatial and temporal analysis, in order to precisely evaluate and forecast trends in road traffic injuries and deaths in older adults, although this would require an enormous effort by governmental agencies from developing countries -a challenge that was recognized in a recent scoping review on interventions to reduce road traffic injuries in Africa [48]; (b) use hospital discharge register data over a long period of time (e.g., 30-days after the crash instead of the police database for deaths recorded within the first 24 hours of the crash); and, (c) include both individual and area-level measures of socioeconomic disadvantage.

## Conclusions

The design of mobility systems for an aging population must consider their needs and abilities. However, research on the impact of road safety measures in objective health outcomes of seniors, such as deaths and injuries, is scarce -especially in developing countries-, and hardly ever within-country socioeconomic inequalities along with the uneven distribution of resources to road safety have received due attention. This study found that in a Latin American country, a traffic law reform aimed at the general population did not lead to a reduction in the rate of road traffic deaths in older adults, that the distribution of the resources to ensure law enforcement were capital to reduce this outcome, and that the rates of deaths in the elderly population due to crashes followed an area-level, social gradient. A social equity approach -addressing the specific needs of the older adults, and the role of the social determinants of health- is needed if road safety measures in developing countries are expected to reduce the burden of traffic fatalities among the elderly.

## Supporting information

S1 Data (CSV)
